# Flow cytometric analysis of Notch1 and Jagged1 expression in normal blood cells and leukemia cells

**DOI:** 10.3892/etm.2012.633

**Published:** 2012-07-04

**Authors:** ERIKO KANAMORI, MAI ITOH, NAOKO TOJO, TAKATOSHI KOYAMA, NOBUO NARA, SHUJI TOHDA

**Affiliations:** 1 Department of Clinical Laboratory;; 2 Laboratory Molecular Genetics of Hematology;; 3 Department of Laboratory Medicine, Tokyo Medical and Dental University, Tokyo, Japan

**Keywords:** notch, jagged, flow cytometry, acute myeloid leukemia, chronic lymphocytic leukemia

## Abstract

Notch1 and its ligand Jagged1 are proteins with important roles in the growth of leukemia cells. Although the detection of Notch1 protein in acute lymphoblastic leukemia cells using immunoblot analysis has been previously reported, the expression patterns of Notch1 and Jagged1 detected by flow cytometry (FCM) in normal blood cells and various leukemia cells have not been well-characterised. In the present study, we examined the expression patterns of Notch1 and Jagged1 in 10 normal blood samples, 8 bone marrow samples, 11 leukemia/lymphoma cell lines and leukemia cells from 22 patients with acute myeloid leukemia (AML), mature T-cell neoplasms or B-cell chronic lymphocytic leukemia (B-CLL) using FCM. The results showed that Notch1 expression is relatively strong in monocytes and granulocytes but weak in lymphocytes. The expression of Notch1 is stronger in bone marrow cells than in the equivalent cells in blood. All the cell lines examined strongly expressed Notch1, and eight cell lines expressed Jagged1. In leukemia cells from patients, four AML samples expressed Notch1 and/or Jagged1. However, three samples expressed neither Notch1 and/or Jagged1 and none of the mature T-cell neoplasm samples expressed either protein. However, all B-CLL samples expressed high levels of both Notch1 and Jagged1. We found that the expression of Notch1 and Jagged1 is detected in various hematological malignancies by FCM. The examination of these proteins is likely to be useful in the characterisation of diseases and individual cases. Examination of these proteins may also be useful in the selection of patients most likely to benefit from novel molecular-targeted therapies using Notch inhibitors in the future.

## Introduction

The fate of hematopoietic stem cells is regulated by Notch signaling. In bone marrow, Notch proteins, such as Notch1, on hematopoietic stem cells are activated by the binding of Notch ligands, such as Jagged1, on stromal cells (1). Notch activation is also involved in the growth of leukemia cells (2), particularly T-cell acute lymphoblastic leukemia (T-ALL) cells. Gene mutations resulting in the activation of Notch1 are present in half of T-ALL cases (3).

Previously, we reported, based on data obtained from immunoblot analyses, that half of the samples of acute myeloid leukemia (AML) cells express Notch1 and/or Jagged1 proteins (4). We also demonstrated that Notch activation induced by Notch ligand stimulation affects the growth of AML cells (5,6). The detection of Notch and Jagged proteins in leukemia cells aids the characterisation of leukemia cases. However, the immunoblot analysis is not suitable for clinical examination since it is time-consuming.

Flow cytometry (FCM) is suitable for clinical examinations since it is currently performed as a routine examination in hospital laboratories. The expression patterns of Notch and Jagged proteins in normal blood cells and leukemia cells have not been well-characterised by FCM. In the present study, we examined the expression of Notch1 and Jagged1 proteins on the surface of normal blood cells, normal bone marrow cells and various leukemia cells.

## Materials and methods

### Cells

Four AML, four T-ALL and three B-cell lymphoma cell lines were used for this study (Table I). NB4 (7) was provided by Dr M. Lanotte (Institut Universitaire d’Hématologie, Paris, France). TMD7 and TMD8 were established in our laboratory. The T-ALL cell lines had *NOTCH1* mutations, and were provided by Dr A. Harashima and Dr K. Orita (Fujisaki Cell Center, Okayama, Japan). The other cell lines were supplied by the Japanese Cancer Research Resources Bank (Tokyo, Japan).

Normal blood samples were obtained from 10 healthy volunteers. Normal bone marrow cells were obtained from bone marrow aspirates with no malignant cells from eight patients with B-cell lymphoma in clinical stage I or II prior to treatment, with informed consent. Blood samples from seven AML patients, five patients with mature T-cell neoplasms and 10 patients with B-cell chronic lymphocytic leukemia (B-CLL) were used with informed consent. The study was approved by the Ethics Review Boards in our University (Tokyo Medical and Dental University, Tokyo, Japan). The profiles of the patients are shown in Table II.

### FCM

The cells were first incubated with Fc receptor saturation reagent (Beckman-Coulter, Brea, CA, USA) for 15 min. The cells were then stained with phycoerythrin (PE)-labeled anti-human Notch1 antibody (clone 527425P; R&D Systems, Minneapolis, MN, USA), fluorescein isothiocyanate (FITC)-labeled anti-human Jagged1 antibody (clone J1G53-3; Enzo Life Science, Plymouth Meeting, PA, USA) or each isotype control antibody. Samples were then incubated with lysing solution (BD Biosciences, Franklin Lakes, NJ, USA), washed with phosphate-buffered saline and analysed using a FACSCalibur cytometer (BD Biosciences). The gates for lymphocytes, monocytes, granulocytes, erythroblasts (in bone marrow samples) and leukemia cells (in leukemia samples) were set in the side scatter vs. forward scatter cytograms or the side scatter vs. CD45 PerCP cytograms. Data were analysed using CellQuest software (BD Biosciences). Data were presented as the percentage increase in mean fluorescence intensity (% increase in MFI), calculated by the equation: [(geometric MFI of relevant antibody - geometric MFI of control)/geometric MFI of control] ×100%. The cut-off 20% increase in MFI was used to divide into positive and negative for convenience.

## Results

### Notch1 and Jagged1 expression in leukemia/lymphoma cell lines

**Figure 1:**
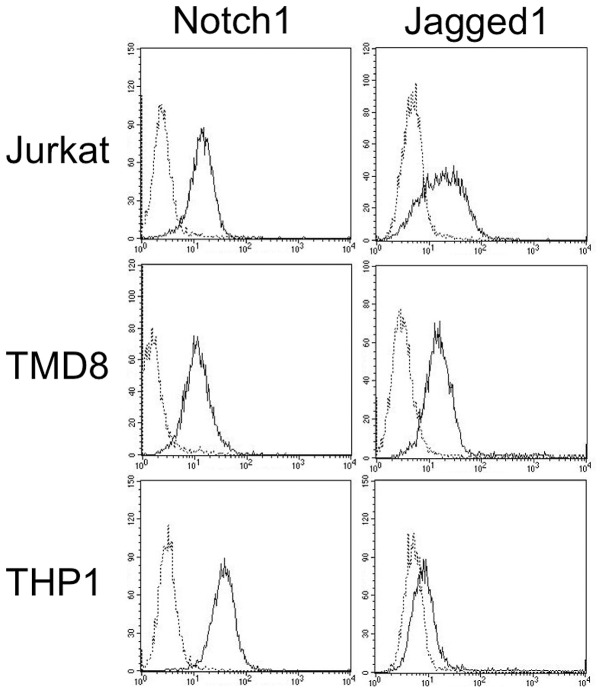
Fluorescence histograms of 3 leukemia/lymphoma cell lines stained with antibodies against Notch1 and Jagged1 (solid lines). The dashed lines represent each isotype control.

We first examined the expression of Notch1 and Jagged1 proteins in 11 leukemia/lymphoma cell lines since we had previously detected the expression of these proteins by immunoblot analysis (4). The fluorescence histograms from the representative cell lines are shown in Fig. 1. The percentage increase in MFI of all the cell lines examined is shown in Table I. The cell lines examined expressed Notch1 protein, and eight cell lines expressed Jagged1 protein. The intensities were found to vary among the cells. Among the four T-ALL cell lines, three cell lines did not express Jagged1.

### Notch1 and Jagged1 expression in normal blood cells and bone marrow cells

**Figure 2:**
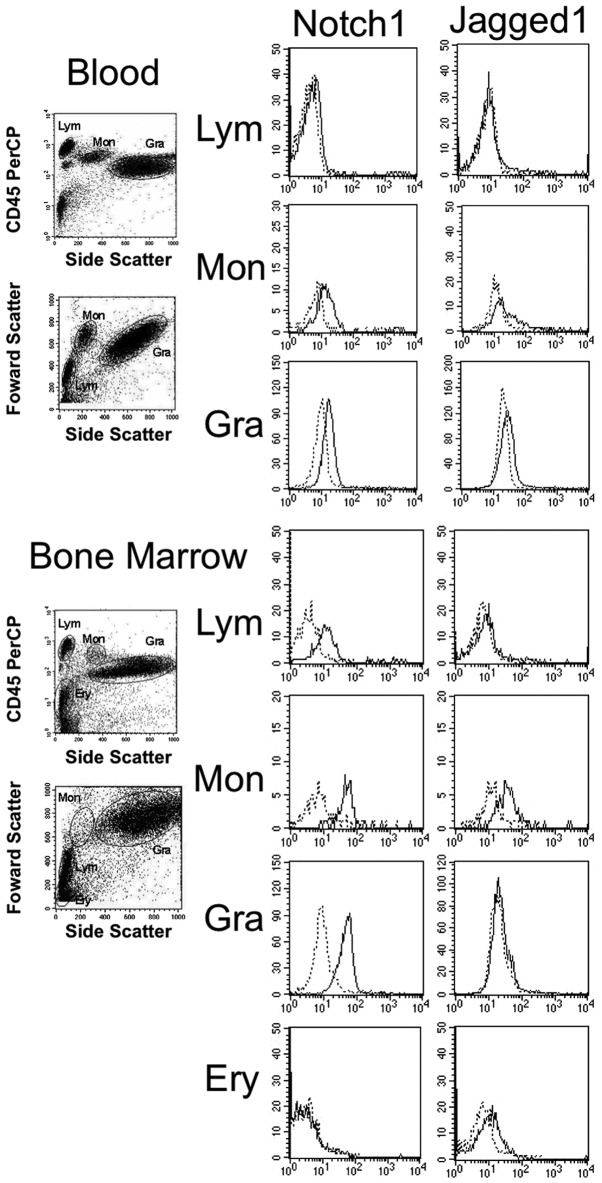
Expression of Notch1 and Jagged1 in normal blood cells (upper panels) and bone marrow cells (lower panels) from representative samples. The gates for lymphocytes (Lym), monocytes (Mon), granulocytes (Gra) and erythroblasts (Ery) in bone marrow samples were set in the side scatter vs. CD45 PerCP cytograms or the side scatter vs. forward scatter cytograms. The solid lines are the histograms of Notch1 and Jagged1. The dashed lines show each isotype control.

The expression of Notch1 and Jagged1 from the representative samples of normal blood and bone marrow aspirates is shown in Fig. 2. The average values of the % increase in MFI of all samples examined are shown in Table III. Notch1 is expressed, in order of increasing intensity, in monocytes, granulocytes, lymphocytes and erythroblasts. The expression of Jagged1 is relatively strong in monocytes. The Notch1 and Jagged1 expression in each cell fraction in blood was found to be weaker than that in the same fraction in bone marrow aspirates.

### Notch1 and Jagged1 in leukemia samples

**Figure 3:**
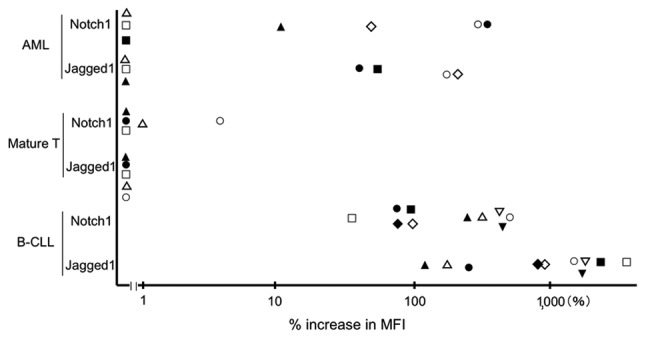
Expression of Notch1 and Jagged1 in leukemia cells from patients as assessed flow cytometry. The blood samples from 7 AML, 5 mature T-cell neoplasm and 10 B-CLL patients were analysed (Table II). Leukemia cells were gated and set according to the same conditions described for Fig. 2. Data are presented as the percentage increase in mean fluorescence intensity (MFI) over isotype control. AML, acute myeloid leukemia; B-CLL, B-cell chronic lymphocytic leukemia.

The intensity of expression of Notch1 and Jagged1 in leukemia cells from 22 patients is shown in Fig. 3. Of seven AML samples, three were found to express both Notch1 and Jagged1, one expressed Jagged1 only and three expressed neither of the proteins. No mature T-cell neoplasm samples expressed either of the proteins. All B-CLL samples expressed both Notch1 and Jagged1. The intensities of the expression of B-CLL cells were much stronger than those of normal lymphocytes.

## Discussion

The present study provides a flow cytometric analysis of Notch1 and Jagged1 expression in normal blood cells and various leukemia cells. Thus far, flow cytometric detection of Notch proteins in monocytes (8), eosinophils (9) and B-ALL cells (10) has been reported. In these studies, permeabilised cells were mainly used for the analysis. Therefore, we aimed to detect Notch1 and Jagged1 in non-permeabilised cells using leukemia/lymphoma cell lines in which Notch1 and Jagged1 expression had been examined by immunoblot analysis. We confirmed that the expression of these proteins was detected in non-permeabilised cells.

We then obtained expression profiles of Notch1 and Jagged1 in each cell fraction in normal blood and bone marrow samples. The results showed that Notch1 expression is strong in monocytes and granulocytes, and weak in lymphocytes. We confirmed that this tendency is also reflected in mRNA expression levels in each cell fraction according to quantitative RT-PCR (data not shown). We also found that the levels of Notch1 and Jagged1 expression are stronger in bone marrow cells than in the equivalent cells in blood. The mechanism of this phenomenon has not yet been identified.

We identified the expression of Notch1 and Jagged1 in various leukemia cells. Since the expression of these genes in T-ALL (3) and B-ALL cells (10) had previously been reported, we focused on AML, mature T-cell neoplasm and B-CLL cells. Certain AML samples expressed Notch1 and/or Jagged1 while other samples expressed neither protein. This observation is similar to the results of our previous study of immunoblot analyses (4). It is well known that Notch1 is crucial in the development of T-ALL (3). By contrast, mature T-cell neoplasm cells did not express Notch1 or Jagged1. This suggests that Notch signaling may not be important for the growth of mature T-cell neoplasm cells. Notably, all the B-CLL samples expressed high levels of both Notch1 and Jagged1. This suggests that Notch signaling is important for the growth of B-CLL cells. This finding is also useful for distinguishing between normal B-lymphocytes and B-CLL cells.

The present study suggests that Notch1 and Jagged1 expression is detected by FCM. To clarify the expression patterns in various types of leukemia, data from more patient samples should be obtained. Subsequently, we predict that the examination of Notch1 and Jagged1 expression is likely to be useful for the characterisation of individual cases, detection of minimal residual diseases and selection of patients that would most benefit from a novel molecular-targeted therapy using Notch inhibitors in the future.

## Figures and Tables

**Table I:** Expression of Notch1 and Jagged1 proteins in leukemia/lymphoma cell lines analysed by flow cytometry.

Lineage	Cell line (type)	Notch1^a^	Jagged1^a^
AML	THP1 (FAB M5)	1,011	69
	TMD7 (FAB M2)	3,024	1,114
	NB4 (FAB M3)	799	23
	HL60 (FAB M2)	1,621	167
T-ALL	Jurkat	445	249
	DND-41	252	13
	ALL-SIL	4,319	0
	KOPT-K1	849	6
B-lymphoma	TMD8 (DLBCL)	539	407
	Daudi (BL)	751	134
	MD901 (BL)	226	422

a Percentage of increase in mean fluorescence intensity over isotype control. FAB, French-American-British classification; DLBCL, diffuse large B-cell lymphoma; BL, Burkitt’s lymphoma; AML, acute myeloid leukemia; T-ALL, T-cell acute lymphoblastic leukemia.

**Table II:** Clinical profiles of the patients.

No.	Lineage	Profile in Fig. 3	Symbol
1	AML	inv(16)	Open circle
2		t(15;17)	Closed circle
3		Myelodysplasia-related changes	Open triangle
4		FAB M0	Closed triangle
5		FAB M2	Open square
6		FAB M2	Closed square
7		FAB M5	Open rhombus
8	Mature T-cell neoplasm	T-prolymphocytic leukemia	Open circle
9		T-prolymphocytic leukemia	Closed circle
10		Adult T-cell leukemia	Open triangle
11		Sézary syndrome	Closed triangle
12		Sézary syndrome	Open square
13–22	B-CLL	Typical CLL	Various

AML, acute myeloid leukemia; B-CLL, B-cell chronic lymphocytic leukemia; FAB, French-American-British classification.

**Table III:** Expression of Notch1 and Jagged1 proteins in normal blood cells and bone marrow cells analysed by flow cytometry.

Source	Cells	Notch1^a^	Jagged1^a^
Blood	Lymphocytes	27	13
	Monocytes	210	82
	Granulocytes	170	40
Bone marrow	Lymphocytes	186	29
	Monocytes	659	298
	Granulocytes	414	81
	Erythroblasts	74	70

a Average percentage increase in mean fluorescence intensity over isotype control of 10 blood samples and 8 bone marrow samples.
